# Persistent Health Issues, Adverse Events, and Effectiveness of Vaccines during the Second Wave of COVID-19: A Cohort Study from a Tertiary Hospital in North India

**DOI:** 10.3390/vaccines10071153

**Published:** 2022-07-20

**Authors:** Upinder Kaur, Sapna Bala, Aditi Joshi, Noti Taruni Srija Reddy, Chetan Japur, Mayank Chauhan, Nikitha Pedapanga, Shubham Kumar, Anurup Mukherjee, Vaibhav Mishra, Dolly Talda, Rohit Singh, Rohit Kumar Gupta, Ashish Kumar Yadav, Poonam Jyoti Rana, Jyoti Srivastava, Shobha Bhat K., Anup Singh, Naveen Kumar P. G., Manoj Pandey, Kishor Patwardhan, Sangeeta Kansal, Sankha Shubhra Chakrabarti

**Affiliations:** 1 Department of Pharmacology, Institute of Medical Sciences, Banaras Hindu University, Varanasi 221005, UP, India; upinder.kaur1@bhu.ac.in; 2 Department of Geriatric Medicine, Institute of Medical Sciences, Banaras Hindu University, Varanasi 221005, UP, India; sapnabala18@gmail.com (S.B.); drrohitbhu@gmail.com (R.S.); rohitkumarguptaji@gmail.com (R.K.G.); dranupbhu@gmail.com (A.S.); 3 Institute of Medical Sciences, Banaras Hindu University, Varanasi 221005, UP, India; aditi.2007.joshi@gmail.com (A.J.); taruni141101@gmail.com (N.T.S.R.); chetanjapur322@gmail.com (C.J.); dr.shubhamkashyap007@gmail.com (S.K.); manurup1997@gmail.com (A.M.); 4 Department of Kriya Sharir, Institute of Medical Sciences, Banaras Hindu University, Varanasi 221005, UP, India; mayankchauhan339@gmail.com; 5 Department of Community Medicine, Institute of Medical Sciences, Banaras Hindu University, Varanasi 221005, UP, India; nikitha72@bhu.ac.in; 6 Department of General Medicine, Institute of Medical Sciences, Banaras Hindu University, Varanasi 221005, UP, India; mvaibhav78@gmail.com; 7 Department of Obstetrics & Gynaecology, Institute of Medical Sciences, Banaras Hindu University, Varanasi 221005, UP, India; talda.dolly041@gmail.com; 8 Center for Biostatistics, Institute of Medical Sciences, Banaras Hindu University, Varanasi 221005, UP, India; ashstatbhu@gmail.com; 9 College of Nursing, Institute of Medical Sciences, Banaras Hindu University, Varanasi 221005, UP, India; jyotirana0669@gmail.com (P.J.R.); jyotichoithram@rediffmail.com (J.S.); 10 Department of Agad Tantra, Institute of Medical Sciences, Banaras Hindu University, Varanasi 221005, UP, India; drbhatshobha@gmail.com; 11 Faculty of Dental Sciences, Institute of Medical Sciences, Banaras Hindu University, Varanasi 221005, UP, India; pgnaveenkumar@gmail.com; 12 Department of Surgical Oncology, Institute of Medical Sciences, Banaras Hindu University, Varanasi 221005, UP, India; manojpandey66@gmail.com

**Keywords:** adverse events following immunization, asthma, inflammatory arthritis, hypothyroidism, long COVID, myocarditis, pharmacovigilance, RAAS blockers

## Abstract

**Background** There is paucity of real-world data on COVID-19 vaccine effectiveness from cohort designs. Variable vaccine performance has been observed in test-negative case-control designs. There is also scarce real-world data of health issues in individuals receiving vaccines after prior COVID-19, and of adverse events of significant concern (AESCs) in the vaccinated. **Methods**: A cohort study was conducted from July 2021 to December 2021 in a tertiary hospital of North India. The primary outcome was vaccine effectiveness against COVID-19 during the second wave in India. Secondary outcomes were AESCs, and persistent health issues in those receiving COVID-19 vaccines. Regression analyses were performed to determine risk factors of COVID-19 outcomes and persistent health issues. **Results**: Of the 2760 health care workers included, 2544 had received COVID-19 vaccines, with COVISHIELD (rChAdOx1-nCoV-19 vaccine) received by 2476 (97.3%) and COVAXIN (inactivated SARS-CoV-2 vaccine) by 64 (2.5%). A total of 2691 HCWs were included in the vaccine effectiveness analysis, and 973 COVID-19 events were reported during the period of analysis. Maximum effectiveness of two doses of vaccine in preventing COVID-19 occurrence was 17% across three different strategies of analysis adopted for robustness of data. One-dose recipients were at 1.27-times increased risk of COVID-19. Prior SARS-CoV-2 infection was a strong independent protective factor against COVID-19 (aOR 0.66). Full vaccination reduced moderate–severe COVID-19 by 57%. Those with lung disease were at 2.54-times increased risk of moderate–severe COVID-19, independent of vaccination status. AESCs were observed in 33/2544 (1.3%) vaccinees, including one case each of myocarditis and severe hypersensitivity. Individuals with hypothyroidism were at 5-times higher risk and those receiving a vaccine after recovery from COVID-19 were at 3-times higher risk of persistent health issues. **Conclusions**: COVID-19 vaccination reduced COVID-19 severity but offered marginal protection against occurrence. The possible relationship of asthma and hypothyroidism with COVID-19 outcomes necessitates focused research. With independent protection of SARS-CoV-2 infection, and high-risk of persistent health issues in individuals receiving vaccine after recovery from SARS-CoV-2 infection, the recommendation of vaccinating those with prior SARS-CoV-2 infection needs reconsideration.

## 1. Introduction

COVID-19 vaccines have been a major deterrent against the raging pandemic. Concerted efforts by several global organizations have resulted in an unprecedentedly rapid development of several vaccine candidates which offer protection against SARS-CoV-2 infection and, more notably, against severe disease and death. However, the efficacy claims of widely used COVID-19 vaccines in the pivotal clinical trials have not been replicated fully in real-world data. The pivotal trials of Pfizer–BioNTech, Moderna, AstraZeneca (manufactured in India as COVISHIELD by the Serum Institute of India), and Bharat Biotech (COVAXIN) were centered around the primary outcome of prevention of PCR-positive SARS-CoV-2 infections [1,2,3,4]. Post-authorization data for most of these leading vaccines have been predominantly derived from test-negative case-control studies [5,6,7]. The test-negative design can control selection and information bias but does not effectively block the bias due to health-seeking behavior which differs between the vaccinated and unvaccinated and is influenced by COVID-19 severity in the individual. Further, the appropriate use of the test-negative design requires baseline matching of the groups with respect to demographic characteristics, prior SARS-CoV-2 infection, comorbidities, symptoms, and other variables [8,9]. The published vaccine effectiveness studies so far have had the limitations of categorizing individuals into cases and controls irrespective of the symptoms, not matching the individuals with respect to exposure to SARS-CoV-2, and excluding individuals with prior SARS-CoV-2 infection or with comorbities [5,6,10]. It is quite likely that the true estimates of vaccine effectiveness may be better calculated in a cohort design while studying a fixed population with similar exposure levels and while considering prior infection and comorbidities [11]. To the best of our knowledge, such studies are lacking.

Further, studies done so far have highlighted the effectiveness rates of vaccines at the time when immune protection against COVID-19 is expected to develop, i.e., 14 days after second dose and 21 days after first dose. Scarce, however, are the studies assessing the epidemiological attributes of COVID-19 and its patterns during the early post-vaccination period. The effect of COVID-19 vaccination on persistent post-COVID health events, sometimes referred to as long COVID, are also worth exploring, particularly in those with a history of natural SARS-CoV-2 infection prior to vaccination. Moreover, though short-term safety analyses of COVID-19 vaccines in controlled settings have provided favorable results, the post-approval period witnessed numerous case reports and series of serious adverse events and adverse events of special interest. These include reports of adverse cardiac events, thrombosis at atypical sites, and new-onset autoimmune diseases [12,13,14]. The issue of long-term safety and adverse events of special interest has not been addressed by the major real-world vaccine effectiveness studies. The incidence and patterns of such adverse events need to be addressed with equal emphasis to provide a better understanding of the benefit–risk ratio of COVID-19 vaccines and to stratify patients at risk of developing adverse events. In this regard, a one-year prospective observational safety study in vaccinated priority groups had been initiated by us in our institute since February 2021 [15].

To address the multiple gaps in knowledge, we conducted a cohort study to include the healthcare workers with almost similar degrees of exposure to SARS-CoV-2 due to occupational reasons. This study centers around two major objectives. It aims to evaluate vaccine performance during the second wave in India, including in the immediate postvaccination period. It also aims to determine the occurrence of adverse events of significant concern in vaccinated individuals. Apart from providing the risk factors of COVID-19 occurrence and severity, for the first time, the study ascertains the real-world effect of timing of vaccination on persistent health issues in individuals who had COVID-19 either prior to or following vaccination.

## 2. Materials and Methods

### 2.1. Study Design and Setting 

This is a cohort study which was conducted during the period of July 2021 to December 2021 in a tertiary university hospital of North India. The study was designed as an extension of an ongoing safety study of vaccinated health care workers [15]. The second wave of the COVID-19 pandemic started from mid-March 2021, peaking around mid-April 2021, and reached the baseline by the end of May 2021 (Supplementary Figure S1). The period from 16 March 2021–31 May 2021 was, hence, primarily selected for the estimation of real-world vaccine effectiveness against COVID-19. However, data was collected until the end of study period (December 2021) and the evaluation of AESCs and persisting health issues was performed until 31 December 2021. The authors UK and SSC had access to the complete data.

### 2.2. Study Participants 

The recruited participants were health care workers. Broadly, they included consultant doctors, teaching faculty, resident doctors, nursing staff, paramedical staff, laboratory personnel, and administrative staff. The participants belonged to modern medical, dental, Ayurvedic (Indian traditional medical), and nursing services. The institute employs nearly 2800+ healthcare workers but the number fluctuates due to residency programs and contractual staff employment. The HCWs available in the institute during working hours were contacted by the study team members as per a planned survey schedule and relevant medical data were collected in a pre-designed case report form. The HCWs who could not be contacted during three visits made on three different days by study team members due to any reason, or those who refused to participate, were not included in the study. Also excluded from the study were those whose COVID-19-related, or COVID-19 vaccination-related details were incomplete. The participants were identified as ‘confirm’ or ‘suspect’ COVID-19 cases as per Ministry of Health and Family Welfare (MoHFW) guidelines, and together labelled as COVID-19 cases [16]. These cases were compared to those with no COVID-19-like events for determination of risk factors of incidence. Severity of COVID-19 was also decided as per MoHFW guidelines [16]. Moderate–severe forms of COVID-19 events were combined into one group and analyzed in determining risk factors of COVID-19 severity. Healthcare workers (HCWs) who were symptomatic during the study period but negative in RT-PCR lab reports were excluded from the analysis. To avoid survival bias, information was collected from each department regarding any deaths of employees during the study period, and family members were contacted to extract the details of vaccination status and mortality of these individuals. 

### 2.3. Ethical Permission

The study started after obtaining ethical permission from the Institute Ethics Committee of the Institute of Medical Sciences, Banaras Hindu University. Written informed consent was obtained from all study participants and legal guardians of the deceased. 

### 2.4. Data Sources/Measurement

All medical data were collected in a pre-designed case report form. The data pertaining to relevant demographics, medical history, concomitant drug history, any history of SARS-CoV-2 infection in the past, COVID-19 vaccination history, adverse events following immunization (AEFIs), and COVID-19-related medical details were collected. For those vaccinated, participants were recruited irrespective of the type of vaccine and center at which the vaccine was received. Case report forms which lacked clarity on COVID-19 or vaccination status were excluded. 

### 2.5. Outcome Measures

#### 2.5.1. Primary Outcomes

The primary outcome of this study was real-world vaccine effectiveness against COVID-19 occurrence during the second wave. Vaccine effectiveness against severity of COVID-19 was also assessed. In addition, the study aimed to predict determinants of both occurrence and severity of disease. 

To generate more robust data on real-world performance of the vaccines, we used multiple analytic designs, in a departure from previously reported literature. Evaluation was performed at various time points as detailed subsequently. 

Three strategies were adopted for analysis of vaccine effectiveness: 

**Strategy A (Assessment of vaccine effectiveness as per standard definitions):** The standard definition of fully vaccinated and partially vaccinated as used in the pivotal clinical trials was used as definition A. Individuals were categorized as ‘2’-dose recipients if they had received their second dose >14 days before the reference date, and as ‘1’-dose recipients if they had received their first dose >21 days before the reference date. The reference date of calculating these time intervals for those developing COVID-19 was either the date of laboratory diagnosis of COVID-19 or the date of the onset of symptoms, whichever was earlier. The reference date of calculating time intervals for those not developing COVID-19 was fixed on 12 April 2021 (peak of second wave in the study population as per Supplementary Figure S1). This strategy was employed because in the case that onset of second wave or end of second wave had been taken as reference date instead of 12 April, there could have been potential underestimation or overestimation of vaccine effectiveness, which we wanted to avoid. Individuals were labelled as recipients of ‘0’ dose if no vaccine dose was received before occurrence of COVID-19 or the date of 12 April 2021.

**Strategy B (Assessment of vaccine effectiveness considering presumed transient immune suppression after first dose of vaccine):** This strategy was considered because there have been some reports of transient immunosuppression after receiving the first dose of the vaccine [17]. To correct for this period, in definition B, participants were labelled as ‘2’-dose recipients if the second dose had been received >14 days before the reference date and were labelled as ‘1’-dose recipients if the first dose had been received at any time before the reference date. As in definition A, reference dates for those developing COVID-19 were either the date of laboratory diagnosis or the date of onset of symptoms, whichever was earlier. The reference date of calculating time intervals for those not developing COVID-19 was fixed on 12 April 2021 (Supplementary Figure S1). The ‘0’-dose group included those who did not receive any vaccine dose before occurrence of COVID-19 or the set date of 12 April 2021. 

**Strategy C (Pure comparison between unvaccinated HCWs and those fully vaccinated before the start of second wave):** This strategy was adopted to give vaccine effectiveness estimates which would be unadulterated by any vaccinations received during the second wave, any hypothetical transient immunosuppression, and any accidental super-spreader events from vaccination centers. 

For this comparison, we considered as one group those HCWs who were fully vaccinated before the start of the second wave, and as comparator group those who were fully unvaccinated until the end of the wave. All participants who had received any dose of vaccine during the period of analysis (period of second wave from 16 March 2021 to 31 May 2021) were excluded from this analysis. For this analysis, since the start of second wave was selected as 16 March 2021, to be considered fully vaccinated, participants must have received their second dose by 1 March 2021. Similarly, unvaccinated individuals were those who had not received any dose of vaccine until 31 May 2021. 

#### 2.5.2. Secondary Outcomes 

Adverse events of significant concern (AESCs) formed the main secondary outcome. Events were graded using the FDA adverse event severity grading scale and categorized under AESC if there was occurrence of any of:

1. Any serious AEFI;
2. Any severe AEFI (FDA grade 3);
3. Any moderate–severe AEFI (FDA grade 2–3) which persisted for ≥7 days;
4. Any moderate AEFI (FDA grade 2) which persisted for ≥4 weeks;
5. Any mild–moderate AEFI (FDA grade 1–2) that persisted for ≥12 weeks.

Causality association was performed for all serious AEFIs using the World Health Organization (WHO) scale of causality assessment.

Other secondary outcomes included health issues in the participants at the time of visit made by the study team. These events were studied individually and those persisting for at least 2 months were explored for any association with history of COVID-19 or COVID-19 vaccine. MedDRA classification was used to assign System Organ Class (SOC) to these events. Broadly, the events were categorized into four groups:

- Vaccine (post-COVID-19)-associated: If health events were reported in those who received COVID-19 vaccine after recovering from natural SARS-CoV-2 infection in the past;
- COVID-19 (post-vaccine)-associated: If events were reported in those who developed COVID-19 after receiving COVID-19 vaccine;
- COVID-19-associated: If events were reported in unvaccinated individuals who developed COVID-19;
- Vaccine-associated: If events were reported in vaccinated individuals with no history of COVID-19 until date of enrollment.

### 2.6. Sample Size

The sample size estimation was based on the major primary outcome of the study, i.e., vaccine effectiveness against COVID-19 occurrence. Considering the 0.6% rate of occurrence of any symptomatic COVID-19 in the vaccinated group and 1.9% rate of occurrence of any symptomatic COVID-19 in the control group (based on Voysey et al.), α of 0.05, and power of 80%, the minimum sample size required for the present study was 2286 [3]. Considering a 5% rate of exclusion of participants because of incomplete information, a minimum sample size of 2400 was required. The data collection was stopped on 31 December 2021, by which time 2765 participants had been enrolled.

### 2.7. Statistical Analysis

Data was represented as frequencies for dichotomous variables and as mean and median values for continuous variables, depending upon skewness. Frequencies and percentages are provided for participants developing AESCs. The chi-square test (or Fisher’s exact test) was applied to assess association between dichotomous variables and occurrence, as well as severity of COVID-19, and also with post-COVID-19 persistent symptoms. Time to occurrence of COVID-19 between vaccinated and unvaccinated groups was compared using the Kaplan–Meier survival analysis. This was followed by the Cox proportional hazard model to predict the risk factors of COVID-19 occurrence after adjusting for potential covariates. To determine risk factors of severity of COVID-19 and risk factors of post-COVID persistent symptoms, binary logistic regression analysis was used. The variables with *p* < 0.05 in unadjusted bivariate analysis were selected for Cox proportional and logistic regression models. 

### 2.8. Role of Funding Source

This study had no funding support.

## 3. Results

### 3.1. Descriptive Data

**Figure 1:**
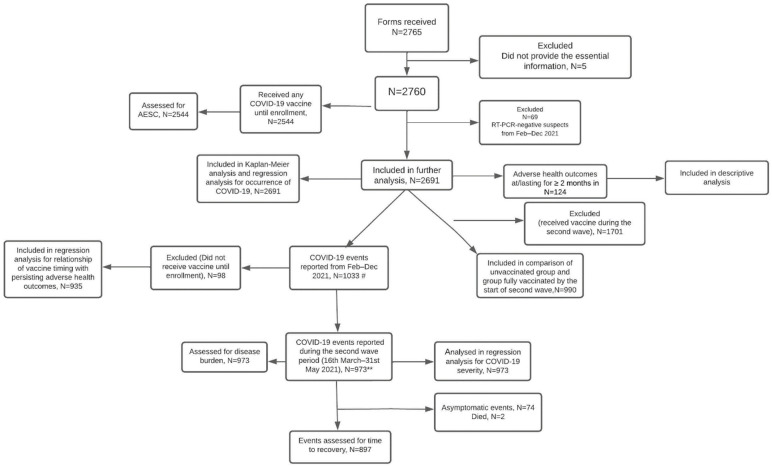
STROBE Flow diagram of selection of participants and steps followed for each analysis. **^#^** 1033 COVID-19 events occurred in 1027 participants ** 973 COVID-19 events occurred in 969 participants.

Overall, medical data were collected from 2765 HCWs. After excluding case report forms of 5 HCWs which lacked essential vaccine-related or COVID-19-related information, a total of 2760 HCWs were enrolled in the study. Figure 1 shows a flowchart of selection of participants and steps followed in each analysis. The mean age of HCWs was 34.9 (±9.9) years (male = 1740, female = 1020). A total number of 2544 HCWs had received COVID-19 vaccines, with COVISHIELD (rChAdOx1-nCoV-19 vaccine) received by 2476 (97.3%) and COVAXIN (inactivated SARS-CoV-2 vaccine) by 64 (2.5%). One HCW each received the Pfizer vaccine (during international travel), the Sputnik vaccine, COVISHIELD followed by COVAXIN, and COVAXIN followed by COVISHIELD. After excluding 69 HCWs who were RT-PCR-negative suspects, 2691 HCWs were included for vaccine effectiveness analysis. A total of 1033 COVID-19 events were identified in them from the period of February to December 2021, out of which 973 events occurred in 969 HCWs in the second wave period or the analysis period for vaccine effectiveness (16 March to 31 May 2021). Four HCWs developed COVID-19 twice in this period. Of these 973 events, 238 were rated as moderate–severe grade (Table 1). 

### 3.2. Main Results

#### 3.2.1. Occurrence of COVID-19

Table 1a shows results of bivariate analysis to determine the association between potential covariates and occurrence of COVID-19. In unadjusted analysis, age, sex, prior history of COVID-19, presence of hypothyroidism, and vaccination status shared a statistically significant association with occurrence of COVID-19 and were selected for Cox proportional hazard analysis. Occurrence of COVID-19 was common in HCWs who were young, females, and had a history of hypothyroidism. No significant difference in COVID-19 occurrence was seen between vaccinated and unvaccinated groups when assessed as per standard definition of vaccination status, i.e., definition A. Interestingly, COVID-19 occurred more commonly in vaccinated individuals compared to unvaccinated when assessed as per definition B which assessed the transient immune suppression after the first vaccine dose and also considered the probability of vaccination centers being super-spreader sites. The occurrence of COVID-19 was less common in HCWs with prior SARS-CoV-2 infection (*p* < 0.001). 

**Figure 2:**
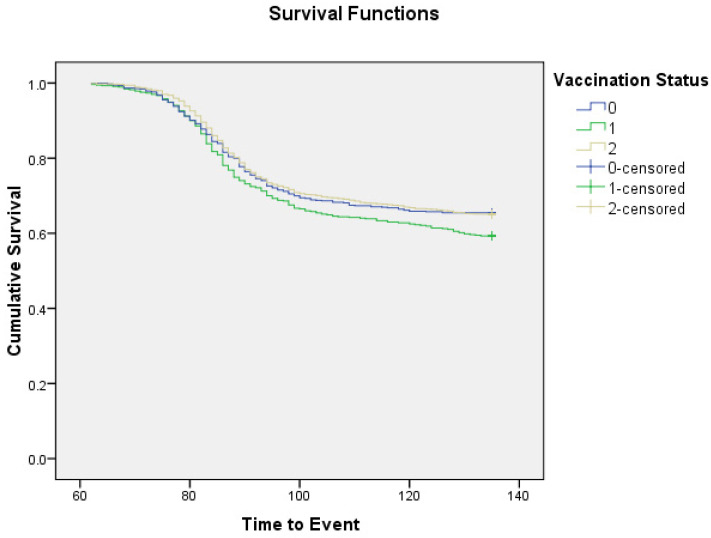
Kaplan–Meier curve showing time to occurrence of event (COVID-19) in ‘1’-dose vaccinated, ‘2‘-dose vaccinated, and unvaccinated groups (as per Strategy B). Analysis period: 16 March 2021 to 31 May 2021.

Figure 2 shows comparison of time to occurrence of event (COVID-19) between vaccinated and unvaccinated groups. Individuals belonging to the ‘1’-dose group developed COVID-19 earlier compared to the unvaccinated and the ‘2’-dose group with statistical significance (*p* = 0.03). After adjusting for potential confounders in the Cox proportional hazard model, age, sex, prior history of COVID-19, and vaccination status emerged as tentative determinants of occurrence of COVID-19 (Table 2a). The risk of occurrence of COVID-19 was nearly 1.45 times greater for those <40 years of age as compared to participants ≥ 40 years, 1.22 times greater for females compared to males, and 1.27 times greater in the ‘1’-dose group compared to the unvaccinated. Prior SARS-CoV-2 infection was an independent protective factor with a 34% lower risk of COVID-19 in this group compared to those with no history of SARS-CoV-2 (*p* < 0.001). 

#### 3.2.2. Severity of COVID-19

Table 1b shows the results of bivariate analysis to determine the association between potential covariates and severity of COVID-19. Pre-existing lung disease and vaccination status were found to be associated with moderate–severe forms of COVID-19 with statistical significance. Disease burden as well as time to recovery from COVID-19 were significantly lower in ‘2’- and ‘1’-dose groups compared to the ‘0’-dose group. Median time to recovery was 10 (7,18) days in the ‘0’-dose group and 7 (5,14) days for the ‘2’-dose group (*p* = 0.002). Disease burden expressed as median number of symptoms was 5 (3,7) in the ‘0’-dose group and 3 (2,5) in the ‘2’-dose group (*p* < 0.001). Vaccine effectiveness for 2 doses (with respect to 0 dose) in reducing severity was 46.4% and 49.1% as per definitions A and B, respectively. The corresponding effectiveness in pure analysis was 51.2% (definition C).

Binary logistic regression analysis showed pre-existing lung disease to be associated with 2.54 times greater odds of moderate–severe COVID-19 (Table 2b). Compared to the unvaccinated, the HCWs in the ‘2’-dose group were at 57% lower risk of suffering from moderate–severe COVID-19.

#### 3.2.3. Persistent Health Issues

A total of 935 COVID-19 events (between February to December 2021), after excluding the events in unvaccinated HCWs, were assessed for relationship between timing of COVID-19 vaccination and health issues persistent for ≥2 months (Figure 1). Table 3 shows association between potential covariates and persistence of adverse health events in HCWs receiving the vaccine before or after COVID-19. With statistical significance, persistent health issues were common in the ≥40 years age group. Vaccinated HCWs with a history of hypothyroidism, inflammatory arthritis, diabetes mellitus, or allergy were more likely to have persistent health issues. Interestingly, a higher percentage of HCWs who received the vaccine after natural COVID-19 (in the year 2021) had persistent health issues compared to those who received the vaccine before COVID-19. Table 4 shows results of logistic regression and validates potential risk factors of persistent health issues in HCWs. After adjusting for potential confounders, presence of hypothyroidism was associated with an approximately 5-times higher risk of persistent health issues, and history of allergy was associated with a 2-times higher risk. Receiving the vaccine after natural SARS-CoV-2 infection of second wave was associated with a nearly 3-times higher risk of persistent health issues. These risk factors were validated even when analysis was extended to involve any SARS-CoV-2 infection of the past, including of the year 2020.

#### 3.2.4. Descriptive Data of Persistent Health Issues

**Figure 3:**
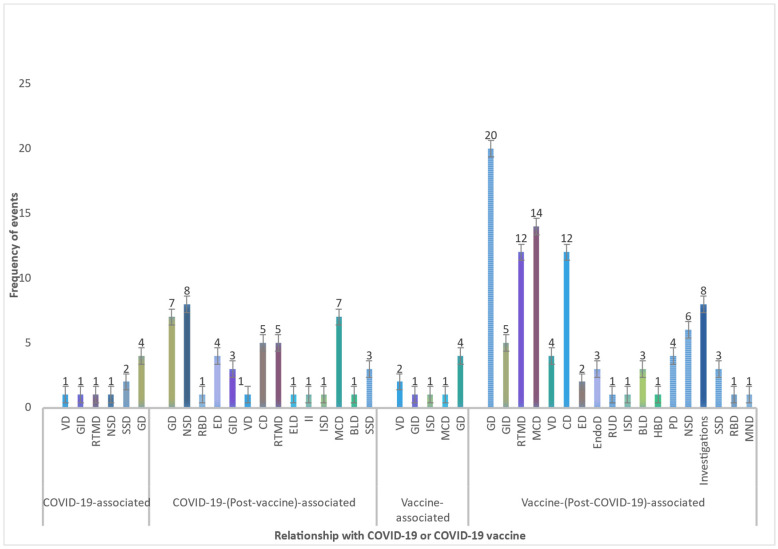
System organ class (SOC) of persistent health issues and their relationship with COVID-19 or COVID-19 vaccine. Analysis period: February 2021 to December 2021. [**MedDRA SOC Abbreviations:** BLD: blood and lymphatic system disorders, CD: cardiac disorders, ED: eye disorders, ELD: ear and labyrinth disorders, Endo D: endocrine disorders, GD: general disorders, GID: gastrointestinal disorders, HBD: hepatobiliary disorders, II: infections and infestations, ISD: immune system disorders, MCD: musculoskeletal and connective tissue disorders, MND: metabolism and nutrition disorders, NSD: nervous system disorders, PD: psychiatric disorders, RBD: reproductive system and breast disorders, RTMD: respiratory, thoracic, and mediastinal disorders, RUD: renal and urinary disorders, VD: vascular disorders, SSD: skin and subcutaneous tissue disorders].

A total of 124 HCWs reported health events which were persisting until date of interview and for at least two months. The MedDRA classification of such events along with their association with COVID-19 or COVID-19 vaccine is shown in Figure 3. A majority (*n* = 74) of the HCWs reporting persistent health events had received the vaccine after natural SARS-CoV-2 infection and belonged to the ‘vaccine (post-COVID-19)’ group. A total of 34 individuals complaining of persistent health issues were in the ‘COVID-19 (post-vaccine)’ group. When classified as per MedDRA, a majority of the persistent health issues (in 124 HCWs) belonged to the SOC of ‘general disorders and administration site conditions’ (*n* = 35) followed by ‘musculoskeletal and connective tissue disorders’ (*n*= 22) and ‘cardiac disorders’ (*n* = 17).

#### 3.2.5. Adverse Events of Significant Concern (AESCs)

Out of the total 2544 HCWs receiving any vaccine at any time until the study’s end date, 33 HCWs developed AESCs (1.3%). A total of seven HCWs developed AEFIs of ‘serious’ grade (0.3%) (details in Table 5). Eight HCWs developed ‘severe’ AEFIs, six had AEFIs of moderate–severe grade that persisted for ≥1 week, eight had ‘moderate’ AEFIs that persisted for ≥4 weeks, and four had mild–moderate AEFIs which lasted for ≥12 weeks. These AESCs persisted with no or partial recovery in 13 HCWs. One HCW had a miscarriage and one died due to a cardiac event. Two HCWs recovered while on new treatment and full recovery was seen in the remaining 16 HCWs. Time to recovery varied from 3–150 days. Of the three deaths reported in our study, one death occurred due to cardiac arrest in a male patient in his 40s with underlying obesity, uncontrolled hypertension, and diabetes. The deceased had received his first dose of vaccine around 1.5 months before the event. The remaining two deaths occurred in unvaccinated individuals, one with underlying hypertension, and the other with underlying diabetes (one confirm COVID-19, one suspect COVID-19).

## 4. Discussion

The design and conduct of this study was preceded and influenced by the preliminary published results of an ongoing prospective study of ours showing high rates (27–46%) of occurrence of COVID-19 in vaccinated priority groups [16]. Nearly 40% breakthrough infection rates were reported by us in doctors, a majority of whom are currently employed in the institute where our study was planned. To make comparisons with the HCWs who were unvaccinated at the time of the pandemic and to generate region-specific vaccine effectiveness data, the study was extended to include other healthcare workers of the institute. 

Vaccine effectiveness rates of 60–80% have been shown in clinical trials and in the majority of the published real-world studies on ChAdOx1-nCoV-19 and inactivated SARS-CoV-2 vaccines [3,4,5,6]. On the contrary, a marginal COVID-19 protection rate (<6%) was observed with COVID-19 vaccines in this study after following the standard definitions of timings of immune protection which have been used in all pivotal trials of COVID-19 vaccines. These wide variations can be explained, to some extent, by the study designs employed in the majority of post-approval vaccine studies. The test-negative case-control designs on which the published studies were based categorize the individuals into cases and controls depending upon the results of laboratory tests. These designs effectively control the selection bias of symptomatology-based traditional case-control studies. Unchecked, however, are the differences in health-seeking behavior due to variable presentations and severity of disease in individuals. The design should not be used in situations where patterns of presentation of disease vary in cases and controls. Baseline characteristics of cases and controls also need to be matched in the absence of which, potential errors are generated in the estimation of vaccine effectiveness rates. Further, since test-negative designs rely only on laboratory tests, the sensitivity and specificity of the laboratory assay becomes a major determining factor in case identification. Many of the post-approval studies have had these limitations.

The maximum vaccine protection observed after excluding the participants who received any dose during the second wave (pure vaccinated vs. unvaccinated cohort analysis performed in Strategy C) was close to 17%. Interestingly, ‘1’-dose recipients showed a higher rate of SARS-CoV-2 infection compared to the unvaccinated. The time to occurrence of COVID-19 in ’1‘-dose recipients was significantly shorter, as corroborated from the findings of the Kaplan–Meier analysis. The high risk of acquisition of disease in ‘1’-dose vaccinated individuals persisted even after adjusting for potential confounders in the Cox proportional hazard model. Compared to the unvaccinated, ’1‘-dose recipients were nearly at 1.3-times higher risk of developing COVID-19. Increased COVID-19 occurrence rate after the first dose has been observed in some other studies and has been linked to the underlying high-risk group of the participants enrolled as well as to the vaccination centers being super-spreading sites of infection [5,6]. However, since a majority of the observed COVID-19 infections in our study were breakthrough, a direct immunomodulatory action of COVID-19 vaccines should also be investigated as a potential cause of suboptimal vaccine protection and increased propensity towards COVID-19. Some evidence, in this regard, is provided by a detailed Chinese study on post-vaccination immune modulation [17]. Interestingly, the exploratory results of a pooled analysis of RCTs suggested a negative vaccine efficacy (−11% to −74%) against asymptomatic COVID-19 within 6–8 weeks of vaccination with the ChAdOx1-nCoV-19 vaccine [18]. 

Among other factors, young individuals <40 years of age and females were observed to be at higher risk of acquiring COVID-19 with respect to comparators. The increased risk of infection acquisition in the young can be attributed to the occupational exposure to SARS-CoV-2, which is high in young health care workers owing to their intensive duty timings compared to the older health care workers. The regression analysis showed prior COVID-19 as a strong independent protective factor associated with lower rates of disease. Nearly 34% lower risk of COVID-19 was observed in individuals with SARS-CoV-2 infection in the past.

With respect to severity of COVID-19, vaccine effectiveness percentages of 46–51% and 13–19% were observed for the ‘2’- and ‘1’-dose groups, respectively, when employing the different analytic strategies. These rates are lower than the severity benefits claimed in controlled settings and some real-world studies on ChAdOx1-nCov-19 vaccines [3,19]. However, the protection offered by vaccines remained statistically significant in regression analysis after adjusting for potential confounders. Compared to the unvaccinated, fully vaccinated individuals were at 57% lower risk of moderate–severe disease. These findings are close to the effectiveness reported by another group from North India [20].

Apart from vaccination status, presence of pre-existing lung disease, particularly asthma, was independently associated with 2.5-times higher odds of moderate–severe COVID-19. In one of our previous studies, around 6-times higher risk of severe forms of COVID-19 was observed in fully vaccinated priority groups with asthma [21]. One possible explanation for increased severity of COVID-19 in health care workers with asthma can be related to their occupation. The health care workers compared to the general population are not able to avoid high-risk situations and, hence, are exposed to the virus for a longer duration. The majority of participants with asthma enrolled in this study had disease of mild–moderate severity and were controlled either on inhaled corticosteroids or systemic leukotriene antagonists. The evidence associating asthma to COVID-19 is conflicting at present. Contrary to expected, asthma was observed to be an underrepresented comorbidity in hospitalized COVID-19 individuals [22]. In a large cohort study using electronic health records of patients in England, mild–moderate asthma (not requiring systemic steroids) was rather not associated with worse clinical outcomes [23]. Only a modest risk of poor COVID-19 outcomes (aHR 1.13) existed with severe asthma. It has been suggested that the Th phenotype of patients should be explored to delineate the relationship of asthma with COVID-19 [24]. 

Several demographic factors and comorbidities are known to be linked with severe forms of COVID-19. These include older age, obesity, kidney disease, diabetes, and cardiovascular disease [25]. No risk was observed with these factors in this study, mainly because of their underrepresentation in the sample of health care workers. 

Another important objective of this study was to predict the determinants of post-COVID-19 persistent health issues, and to shed light on the safety profile of COVID-19 vaccines after a natural SARS-CoV-2 infection. The effect of COVID-19 vaccines on persistent health issues when administered after natural COVID-19 has been largely unexplored. After adjusting for potential confounders, presence of hypothyroidism was associated with a more than 5-times higher risk of post-COVID persistent health issues, compared to those with euthyroid state. A nearly 2-times higher risk was evident in individuals with a history of allergy to any stimuli. Interestingly, a close to 3-times higher risk of post-COVID persistent health issues was observed in individuals receiving the vaccine after past natural SARS-CoV-2 infection in 2021, compared to those who received the vaccine before getting the infection. Presence of inflammatory arthritis was also associated with higher risk of post-COVID persistent symptoms, though the confidence intervals varied widely and the overall number of individuals with immune-mediated arthritis was small. The associations of all these proposed risk factors were corroborated in a separate regression analysis extended to involve any SARS-CoV-2 infection in the past, including of the year 2020 (data not shown). In light of much new evidence that vaccine-induced immunity is short-lasting, vaccinating those recovered from natural infection needs close scrutiny.

A detailed safety analysis was performed in individuals with health issues persistent for ≥2 months. A majority (60%) of them had received the vaccine post-recovery from natural SARS-CoV-2 infection. Nearly 27% of persistent health issues were related to developing COVID-19 post-vaccination. Adverse events of significant concern (AESCs) developed in 33 participants, giving an AESC rate close to 1.3%. Serious AEFIs occurred in 0.3% of HCWs. Considering AESCs with ‘probable’ causality association with the vaccine, the incidence of cardiac events following COVID-19 vaccines and that of severe hypersensitivity reactions were about 1 per 2544 vaccinees, which is higher than what has been claimed by vaccine manufacturers. Of the three deaths reported, one occurred due to cardiac arrest in a partially vaccinated individual with multiple comorbidities and the remaining two deaths occurred in unvaccinated comorbid individuals, possibly due to COVID-19. The partially vaccinated HCW had background risk factors of poor cardiac outcomes, and the event was considered to have ‘Unlikely’ causality association with the vaccine. With small number of fatalities overall, no statistically relevant conclusions could be made on the mortality benefits of COVID-19 vaccination.

The study adds new information to the existing evidence. For the first time, through bypassing the shortcomings of test-negative case-control designs, the vaccine effectiveness estimates have been generated from a better, albeit cumbersome, cohort design with adjustment for confounders. It is also the first of its kind study to ascertain the effect of COVID-19 vaccines received after natural COVID-19 on persistent adverse health outcomes. Apart from the effectiveness rates, estimates and types of adverse events of significant concern and persistent health issues would guide researchers and policy makers to revisit the benefit–risk ratio of vaccines in mass rollout. The development of safer vaccine alternatives and regimes may also be stimulated by these data. 

### Limitations

The possibility of recall bias exists in the study due to its retrospective design. However, the participants being healthcare workers, the information may be considered mostly reliable. The reference date for those not developing COVID-19 was selected as the time COVID-19 cases peaked in the institute. This was done so as to prevent potential under- or overestimation of effectiveness of COVID-19 vaccines. However, the analysis conducted using strategy C neutralized the adulterating effects of vaccination received during the time of the second wave, thus, providing a closer estimate of vaccine effectiveness. Future studies choosing different reference dates with respect to the onset and end of the wave can be designed to generate point-specific estimates. The study being conducted in health care workers, the observation of high rates of COVID-19 in the entire sample should be generalized with caution as risk of exposure to respiratory viruses is high in health care workers compared to the general population. As the study is based on a predominantly healthy younger population with limited representation of comorbidities such as heart disease, diabetes, hypothyroidism, and lung disease, the results may not be extrapolated to the general Indian population with different demographics and varying disease prevalence rates. Despite the risk of COVID-19 severity observed with lung disease and association of AESCs with hypothyroidism, and inflammatory arthritis, the overall number of participants with these comorbidities was small. Future studies with better representation of comorbidities would provide better insights on the possible disease-mediated modulation of COVID-19 outcomes and adverse events following vaccines. Likewise, the majority of the vaccinees in this study had received COVISHIELD and only <3% had received COVAXIN. Thus, findings of the study are more focused towards COVISHIELD and cannot be extrapolated to COVAXIN. Because of the small number of death events, and no autopsy details available in this questionnaire-based study, not much interpretation can be made in regard to the mortality benefits with COVID-19 vaccines. Due to funding issues, SARS-CoV-2 anti-spike antibody titer was not estimated at any time during the study, resulting in a possibility of missing out on prior asymptomatic infections. However, such cases may be presumed to have a uniform distribution across the study population. Some asymptomatic infections during the study period may have also been missed, as RT-PCR tests were not mandated for the asymptomatic as per existing guidelines. However, the study population being of healthcare workers, PCR testing for work safety reasons and on developing any symptoms was regular among participants. Though the Delta variant was believed to be the predominant strain in the affected region as previously reported, genome sequencing was not done in any of the COVID-19 cases and, hence, SARS-CoV-2 variant-specific information cannot be provided [16]. Furthermore, though the study detected serious adverse events at higher-than-expected rates, the exact incidence of rare but serious events needs confirmation from larger sample studies. This study being conducted in a localized region of North India provides a region-specific vaccine performance and, therefore, might have more of local impact on future vaccination policies. In some behavior assessment studies, vaccines have been accepted as an effective preventive measure against COVID-19 by specific high-risk groups such as elderly [26]. Future cohort studies with adequate enrollment of elderly and different ethnicities are recommended to generate risk group-specific evidence on vaccine performance.

## 5. Conclusions

COVID-19 vaccination provided a marginal protection against the occurrence of COVID-19 and a modest protection against the severity of disease. Compared to the unvaccinated, a high risk of occurrence of COVID-19 was observed in participants receiving ‘1’ dose of vaccine. Previous infection by SARS-CoV-2 acted as an independent protective factor against COVID-19 occurrence. Pre-existing lung disease, mainly asthma, independently enhanced the risk of moderate–severe COVID-19 in health care workers and necessitates a focused study of COVID-19 vaccines in individuals with asthma. 

Receiving any dose of vaccine after natural SARS-CoV-2 infection was associated with higher risk of persistent health issues. With considerably lower protection against COVID-19 than predicted from controlled settings and a higher risk of chronic health issues in those vaccinated after natural SARS-CoV-2 infection, the authors suggest that the concept of vaccinating recovered individuals may need closer scrutiny. It may not be beneficial in the light of multiple breakthrough infections and short-lasting immunity post-vaccination. Vigilance for prolonged health events is warranted in individuals with hypothyroidism and history of allergy, and those receiving any COVID-19 vaccine after natural SARS-CoV-2 infection. Patients of inflammatory arthritis also need to be monitored for long-term health events post-COVID-19 vaccine. Future vaccination policies might consider the history of prior natural COVID-19 in the individual, and history of significant adverse events with any dose of vaccine and incorporate in the label a need for special monitoring of groups at high risk of adverse outcomes. The incidence of severe hypersensitivity reactions and myocarditis seems to be higher than what has been claimed, and warrants larger studies focused on long-term vigilance of vaccines. Regulatory authorities might also encourage transparent post-marketing surveillance for COVID-19 vaccines and boosters. 

## Figures and Tables

**Table 1:** Baseline characteristics of study population and bivariate analysis to determine risk factors of COVID-19 occurrence and severity in health care workers during the second wave of pandemic (between 16 March and 31 May 2021).

1a				1b		
	Participants (N = 2691)	COVID-19 Cases, n (%)	*p*-Value (Effect Size)	COVID-19 Events (N = 973) *	Events of Moderate– Severe Grade	*p*-Value (Effect Size)
**Age (years)**			<0.001 (OR 1.5)			
<40	2009	773 (38.5)		777	185 (23.8)	0.35
≥40 (reference)	682	196 (28.7)		196	53 (27)	
**Sex**			<0.001 (OR 1.4)			
Male (reference)	1699	567 (33.4)		569	134 (23.6)	0.43
Female	992	402 (40.5)		404	104 (25.7)	
**Body mass index (kg/m^2^)** **						
≥25	1056	397 (37.6)	0.23	398	106 (26.6)	0.19
<25	1633	572 (35)		575	132 (23)	
**Diabetes mellitus**						
Yes	154	45 (29.2)	0.07	45	11 (24.4)	0.99
No	2537	924 (36.4)		928	227 (24.5)	
**Hypertension**						
Yes	201	74 (36.8)	0.80	74	18 (24.3)	0.97
No	2490	895 (35.9)		899	220 (24.5)	
**Heart disease**						
Yes	28	7 (25)	0.22	7	3 (42.9)	0.26
No	2663	962 (36.1)		966	235 (24.3)	
**Lung disease**						0.03 (OR 2.3)
Yes	80	26 (32.5)	0.51	26	11 (42.3)	
No (reference)	2611	943 (36.1)		947	227 (24)	
**Hypothyroidism**			0.03 (OR 1.5)			
Yes	107	49 (45.8)		49	15 (30.6)	0.30
No (reference)	2584	920 (35.6)		924	223 (24.1)	
**Inflammatory arthritis**						
Yes	14	6 (43)	0.59	6	3 (50)	0.14
No	2677	963 (36)		967	235 (24.3)	
**History of allergy**						
Yes	342	137 (40.1)	0.09	137	34 (24.8)	0.92
No	2349	832 (35.4)		836	204 (24.4)	
**Use of RAAS blockers**						
Yes	111	43 (38.7)	0.54	43	10 (23.3)	0.85
No	2580	926 (36)		930	228 (24.5)	
**Prior history of COVID-19** Yes No (reference)	387 2304	104 (26.8) 865 (37.5)	<0.001 (OR 1.6)	105 868	32 (30.5) 206 (23.7)	0.13
**Type of Vaccine (N = 2476)** COVISHIELD COVAXIN COVISHIELD/COVAXIN COVAXIN/COVISHIELD Pfizer	2412 61 1 1 1	859 (35.6) 16 (26.2) 1 1 0	0.16	863 16 - - -	195 (22.6) 4 (25) - - -	0.07
**Vaccination status** **(Definition A)** 0 dose 1 dose 2 doses	935 394 1362	346 (37) 148 (37.6) 475 (34.9)	0.45	348 149 476	113 (32.5) 42 (28.2) 83 (17.4)	<0.001(Cramer’s V 0.16)
**Vaccine effectiveness (Definition A)** 2 versus 0 1 versus 0	5.7% −1.6%				46.4% 13.2%	
**Vaccination status** **(Definition B)** 0 dose 1 dose 2 doses	759 570 1362	262 (34.5) 232 (40.7) 475 (34.9)	0.03(Cramer’s V 0.05)	263 234 476	90 (34.2) 65 (27.8) 83 (17.4)	<0.001(Cramer’s V 0.17)
**Vaccine effectiveness (Definition B)** 2 versus 0 1 versus 0	−1.1% −18%				49.1% 18.7%	
**Pure analysis** 0 dose 2 doses (reference)	500 490	227 (45.4) 184 (37.6)	0.01 (OR 1.4)	227 184	81 (35.7) 32 (17.4)	<0.001 (OR 2.6)
**Vaccine effectiveness** **(Pure analysis)** 2 versus 0	17.2%				51.2%	

1a: Bivariate analysis for risk factors of occurrence; 1b: Bivariate analysis for risk factors of moderate–severe COVID-19. * In four participants, COVID-19 occurred two times during the study period. ** Body weight information was not provided by two participants. Abbreviations: COVID-19, coronavirus disease-2019; RAAS, renin angiotensin aldosterone system; Effect size mentioned as odds ratio only for those variables with significant *p*-value (reference category with respect to which odds are calculated is mentioned for each variable in bracket); For variable with more than two categories and with significant *p*-value, Cramer’s V is mentioned instead of odds ratio.

**Table 2:** Regression analyses to determine tentative risk factors for occurrence and severity of COVID-19 during the second wave of pandemic between 16 March and 31 May 2021.

2a			2b		
Tentative Risk Factors (*n* = 2691)	aHR	*p*-Value	Tentative Risk Factors (*n* = 973 COVID-19 Events)	aOR	*p*-Value
**Age (years)** <40 ≥40 (Reference)	**1.45** (1.23–1.69)	**<0.001**	**Pre-existing lung disease** Yes No (Reference)	**2.54** (1.13–5.7)	**0.02**
**Sex** Female Male (Reference)	**1.22** (1.07–1.39)	**0.004**			
**Prior history of COVID-19** Yes No (Reference)	**0.66** (0.54–0.81)	**<0.001**	**Vaccination status** ** 2 doses 1 dose 0 dose (Reference)	**0.43** (0.31–0.60) 0.83 (0.54–1.26)	**<0.001** 0.38
**Hypothyroidism** Yes No (Reference)	1.34 (0.99–1.8)	0.055			
Vaccination status * 2 doses 1 dose 0 dose (Reference)	**1.1** (0.92–1.26) 1.27 (1.10–1.52)	0.34 **0.007**			

2a: Cox-proportional hazard model for risk factors of occurrence; 2b: Binary logistic regression analysis for risk factors of moderate–severe COVID-19. aHR: adjusted hazard ratio; aOR: adjusted odds ratio. *: as per definition B (*p* < 0.05 in unadjusted bivariate analysis, only for definition B). **: as per definition A. With both definitions of vaccination status showing *p* < 0.05 in unadjusted bivariate analysis, the standard definition (definition A) was chosen for logistic regression analysis. Similar statistical results were seen even with definition B.

**Table 3:** Bivariate analysis to determine risk factors for persisting health issues in health care workers (*n* = 935 COVID-19 events) with history of COVID-19 vaccination before or after COVID-19 between February to December 2021.

Risk Factor	COVID-19 Events	Persistent Adverse Health Outcomes, N (%)	*p*-Value (Effect Size)
**Age (years)**			**0.04 (OR 1.6)**
<40 (reference)	740	69 (9.3)	
≥40	195	28 (14.4)	
**Sex**			0.12
Male	560	51 (9.1)	
Female	375	46 (12.3)	
**Body mass index (kg/m^2^)**			0.56
≥25	382	37 (9.7)	
<25	553	60 (10.8)	
**Diabetes mellitus**			**0.04 (OR 2.2)**
Yes	46	9 (19.6)	
No (reference)	889	88 (9.9)	
**Hypertension**			0.69
Yes	68	8 (11.8)	
No	867	89 (10.3)	
**Heart disease**			0.40
Yes	6	0 (0)	
No	929	97 (10.4)	
**Lung disease**			0.22
Yes	29	5 (17.2)	
No	906	92 (10.2)	
**Hypothyroidism**			**<0.001 (OR 4.8)**
Yes	49	16 (32.7)	
No (reference)	886	81 (9.1)	
**Inflammatory arthritis**			**0.004 (OR 26.7)**
Yes	4	3 (75)	
No (reference)	931	94 (10.1)	
**History of allergy**			**0.008 (OR 2)**
Yes	137	23 (16.8)	
No (reference)	798	74 (9.3)	
**Vaccine received after COVID-19 recovery**			**<0.001 (OR 2.5)**
Yes	331	54 (16.3)	
No (reference)	604	43 (7.1)	
**Type of Vaccine**			**0.21**
COVISHIELD	916	94 (10.3)	
COVAXIN	17	2 (11.8)	
COVISHIELD/COVAXIN	2	1 (50)	

Effect size mentioned as odds ratio only for those variables with significant *p*-value (with reference category mentioned for each variable in bracket).

**Table 4:** Regression analysis to determine risk factors for persisting health issues in health care workers with history of COVID-19 vaccination before or after COVID-19 between February to December 2021.

Tentative Risk Factor	Adjusted Odds Ratio	*p*-Value
**Vaccination after COVID-19** Yes No (reference)	**2.8** (1.8–4.4)	**<0.001**
**Age (years)** <40 ≥40 (reference)	0.7 (0.4–1.1)	0.14
**Diabetes mellitus** Yes No (reference)	1.1 (0.4–2.7)	0.83
**Inflammatory arthritis** Yes No (reference)	30 (3–304)	**0.004**
**History of allergy** Yes No (reference)	**2** (1.2–3.4)	**0.01**
**Hypothyroidism** Yes No (reference)	**5.3** (2.6–10.5)	**<0.001**

**Table 5:** Serious AEFIs following vaccination.

Age/Sex	Comorbidity	Type of Vaccine	Time of AEFI since COVID-19 Vaccine	Description of AEFI	Outcome	Causality
29 years/Female	History of allergy, Polycystic ovarian disease	COVAXIN	Within 24 h of first dose	Fever, severe vomiting, and diarrhoea within 24 h of first dose, requiring hospitalization	Recovered in 5 days	Probable
37 years/Female	Hypothyroidism	COVISHIELD	Within 24 h of first dose	Tingling, dizziness, palpitations, heaviness in chest, tachycardia, and fluctuating blood pressure. On admission, blood pressure 150/80 mm Hg, heart rate 130/min, remaining vitals stable and routine blood investigations including cardiac enzymes were normal.	Recovered in 4 days	Possible
38 years/Female	Diabetes mellitus	COVISHIELD	Within three months of second dose	Miscarriage	NA	Possible
32 years/Female	--	COVISHIELD	Within 24 h of first dose	Abdominal distress and severe diarrhoea requiring emergency room visit	Recovered in 5 days	Possible
45 years/Male	Diabetes mellitus, hypertension, obesity	COVISHIELD	Within 8–10 weeks of first dose	Cardiac arrest	Died, NA	Unlikely
39 years/Female	--	COVISHIELD	Within 24 h of second dose	Rashes, breathlessness, drowsiness, hypophonia, tachycardia, mild headache. Rash also followed first vaccine dose.	Recovered fully in 3–4 days	Probable
47 years/Female	Hypothyroidism, hypertension, old lung cyst, RT-PCR positive for SARS-CoV-2 three and a half months before vaccination	COVISHIELD	Within seven days of first dose	Fever, nausea, chest pain, dyspnoea, palpitation, difficulty in talking, increased erythrocyte sedimentation rate, eosinophilia (6.2%). Routine kidney and liver function tests were normal. Cardiac enzymes done after 1 week of symptom onset normal, 2D Echocardiography normal. Cardiac magnetic resonance imaging was suggestive of myocarditis.	Recovered in 30 days	Probable

Abbreviations: AEFI, adverse event following immunization; COVID-19, coronavirus disease-2019; NA, not applicable.

## Data Availability

Associated data may be made available by the corresponding author on reasonable request.

## Supplementary Materials

The following supporting information can be downloaded at: https://www.mdpi.com/article/10.3390/vaccines10071153/s1, Figure S1. Date-wise COVID-19 case counts during second wave in study population.
