# Supplementary figures and images for: Persistent Health Issues, Adverse Events, and Effectiveness of Vaccines during the Second Wave of COVID-19: A Cohort Study from a Tertiary Hospital in North India
Source: Vaccines (Basel). 2022 Jul 20;10(7):1153. doi: 10.3390/vaccines10071153 (PMC9319407; doi:10.3390/vaccines10071153)

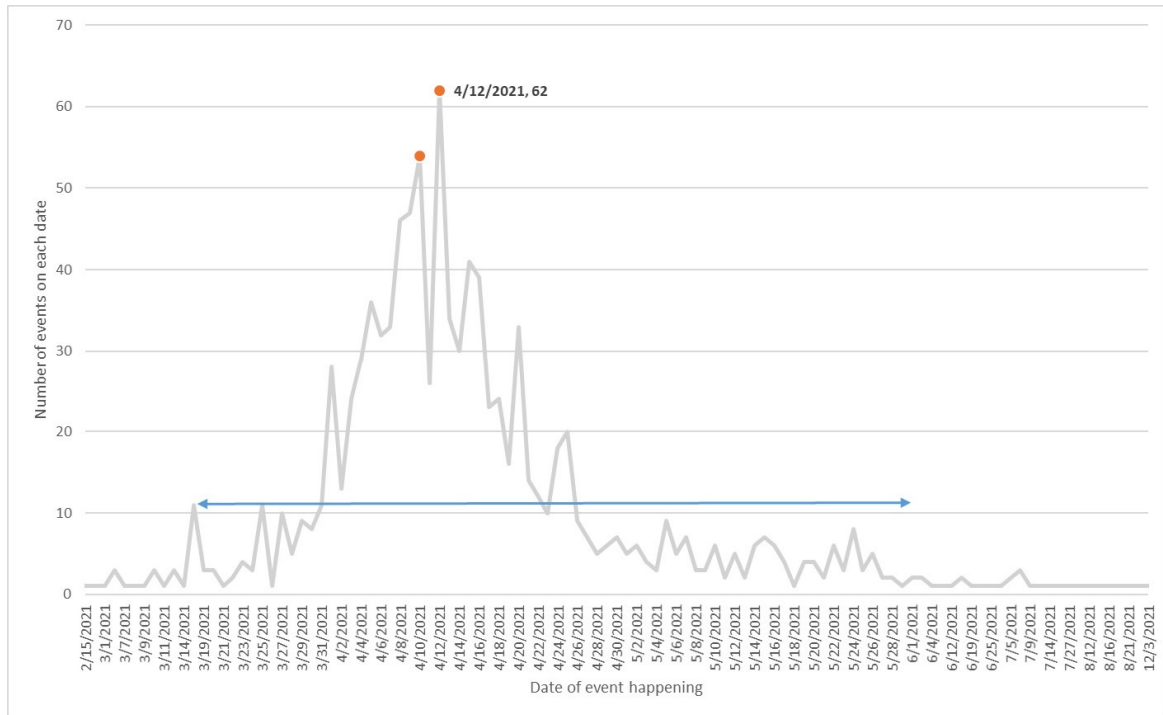

**Figure S1.** Date-wise COVID-19 case counts during second wave in study population.

Supplement: Supplementary file 1 [file vaccines-10-01153-s001.zip › vaccines-1771675-supplementary.pdf]
